# Supplementary material for: Microglial Cytokines Induce Invasiveness and Proliferation of Human Glioblastoma through Pyk2 and FAK Activation
Source: Cancers (Basel). 2021 Dec 7;13(24):6160. doi: 10.3390/cancers13246160 (PMC8699228; doi:10.3390/cancers13246160)
Supplement: Supplementary file 1 [file cancers-13-06160-s001.zip › cancers-1484034-supplementary.pdf]

cancers

MDPI

Supplementary Materials: Microglial cytokines induce invasiveness and proliferation of human glioblastoma through Pyk2 and FAK activation

Rebeca E. Nuñez, Miguel Mayol del Valle, Kyle Ortiz, Luis Almodovar, Lilia Kucheryavykh

Table S1. List of primers, used in RT-PCR.

Protein name	Gene name	Catalog #	Source
Pyk2	PTK2B	QT00073402	Qiagen
FAK	PTK2	QT00057687	Qiagen
EGFR	EGFR	QT00085701	Qiagen
PDGFRα	PDGFRA	10025636	Bio-Rad
PDGFRβ	PDGFRB	QT00082327	Qiagen
NGFR	NGFR	QT 00056756	Qiagen
IL-6R	IL6R	10025636	Bio-Rad
CXCR1	CXCR1 (IL8RA)	QY00212919	Qiagen
CXCR4	CXCR4	10025636	Bio-Rad
CCR5	CCR5	QT01336601	Qiagen
NGF	NGFB	10025636	Bio-Rad
EGF	EGF	10025636	Bio-Rad
PDGFα	PDGFA	10025636	Bio-Rad
PDGFβ	PDGFB	10025636	Bio-Rad
IL-6	IL6	10025636	Bio-Rad
IL-8	CXCL8	10025636	Bio-Rad
CCL5	CCL5	10025636	Bio-Rad
CXCL12	CXCL12 (SDF1A)	10041595	Bio-Rad
GAPDH	GAPDH	Forward 174079688<br>Reversed 174079689	Integrated DNA Technologies

a

Patient	Sex	Age	Tumor location	IDH mutation	Tumor grade
1	F	59	Right Frontal lobe	wt	IV
2	M	67	Left intra-axial	wt	IV
3	F	62	Right temporal	wt	IV

b

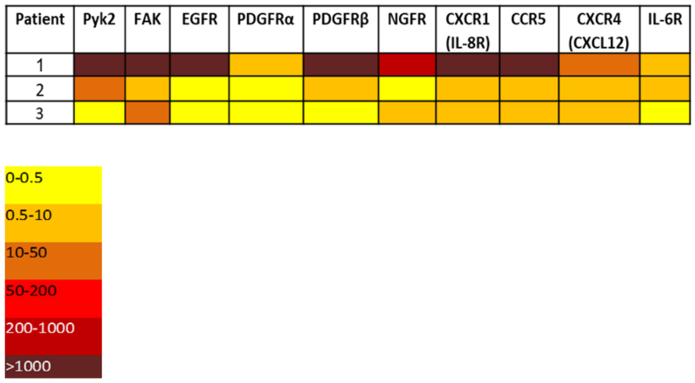

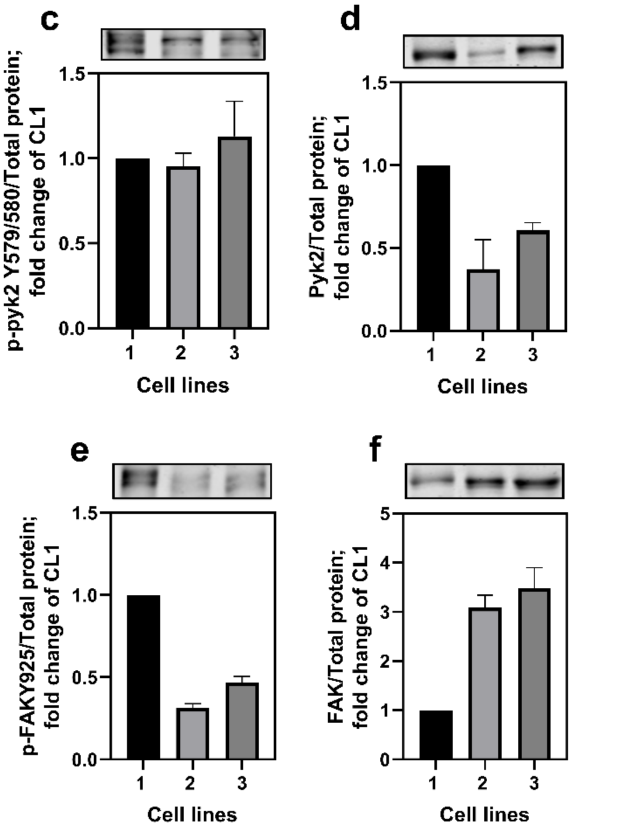

Figure S1. Characterization of GBM-derived primary cells (CL1, CL2, and CL3). (**a**) Patient data. (**b**) Pyk2, FAK and selected cytokine and chemokine receptors' gene expression levels in GBM derived primary cells, detected by RT-PCR. (**c–f**) Pyk2 and FAK protein expression and phosphorylation levels in GBM derived primary cell lines, detected by western blot. Representative western blots and quantitative results for total and phosphorylated Pyk2 (Y579/580) and FAK (Y925) are presented. The degree of phosphorylation was calculated as the ratio of phosphorylated Pyk2 or FAK to the total loaded protein and normalized to the control for each kinase. The values are shown as means ± SD of three repetitions per cell line.

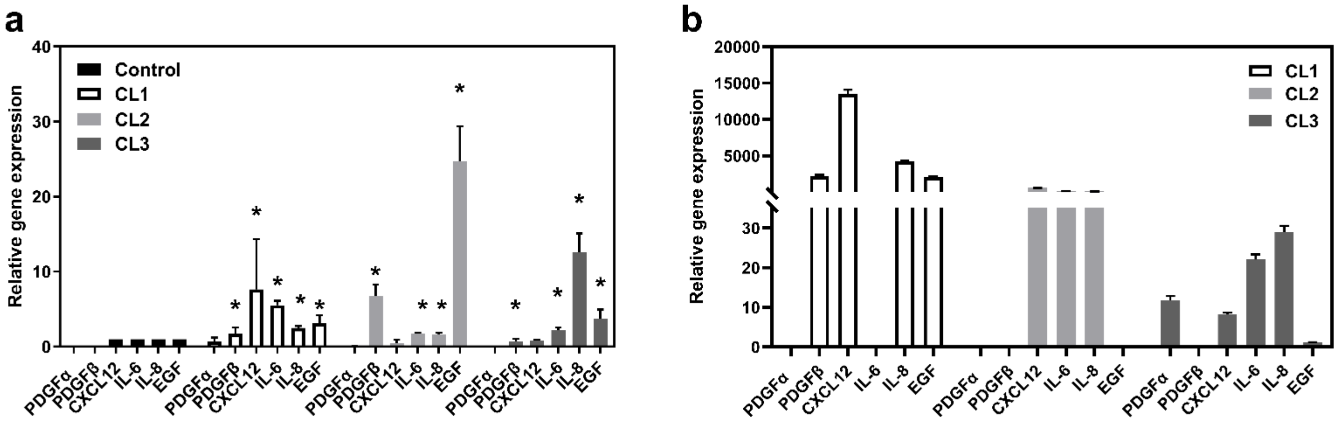

Figure S2. Selected cytokines and chemokines relative RNA expression levels, identified with use of RT-PCR in (**a**) control HMC3 microglia and microglia treated with medium conditioned from CL1, CL2 or CL3 glioma cells and (**b**) in GBM derived primary cells (CL1, CL2, and CL3).

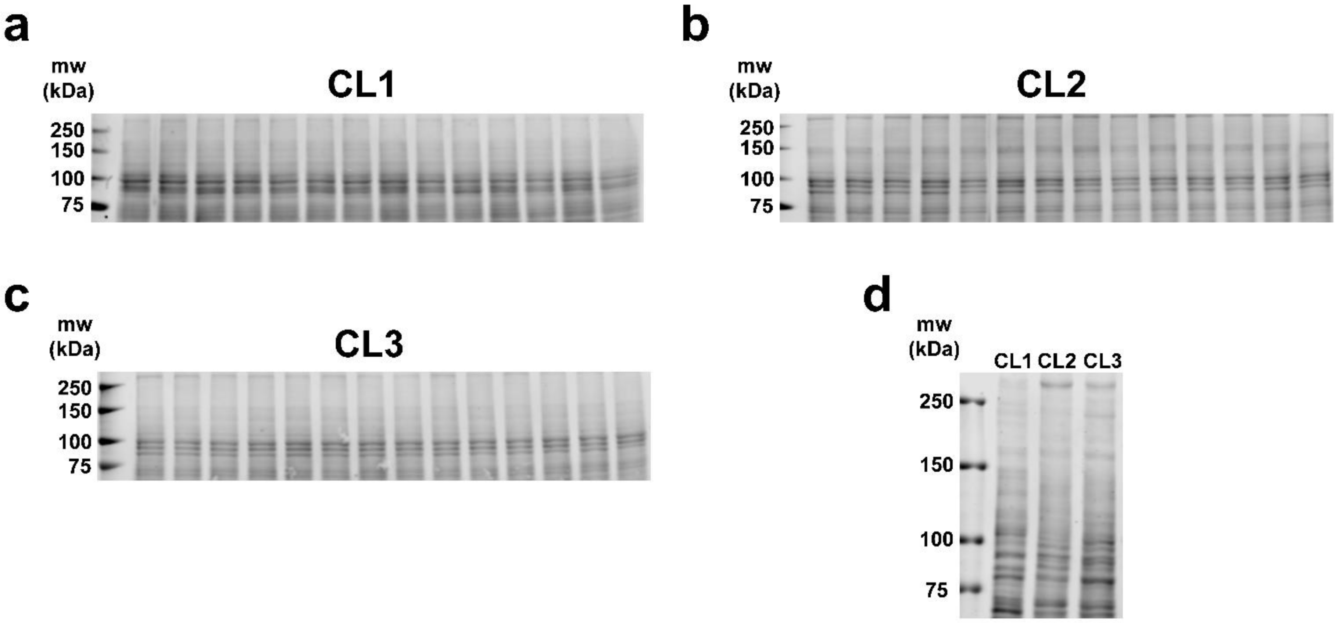

Figure S3. Membranes stained for total protein as a loading control for western blot identification of Pyk2 and FAK. Membranes were stained with REVERT Total Protein Stain from LI-COR Biotechnology. (**a**) CL1, (**b**) CL2, (**c**) CL3 membranes in support of Figure 2. (**d**) CL1, CL2, and CL3 membranes in support of Figure S1c–f.

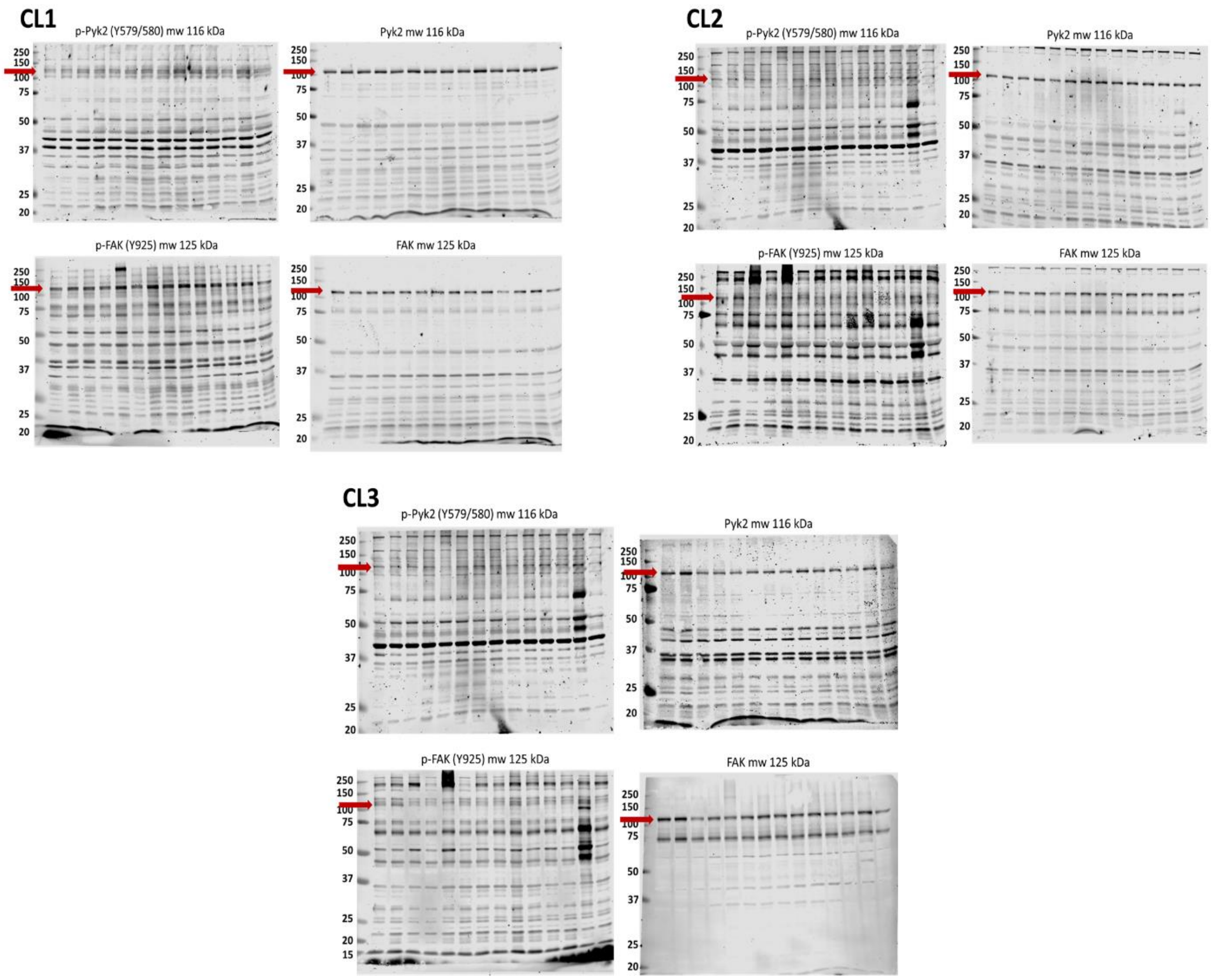

Figure S4. Uncropped western blot membranes for identification of Pyk2 and FAK in support of Figure 2.

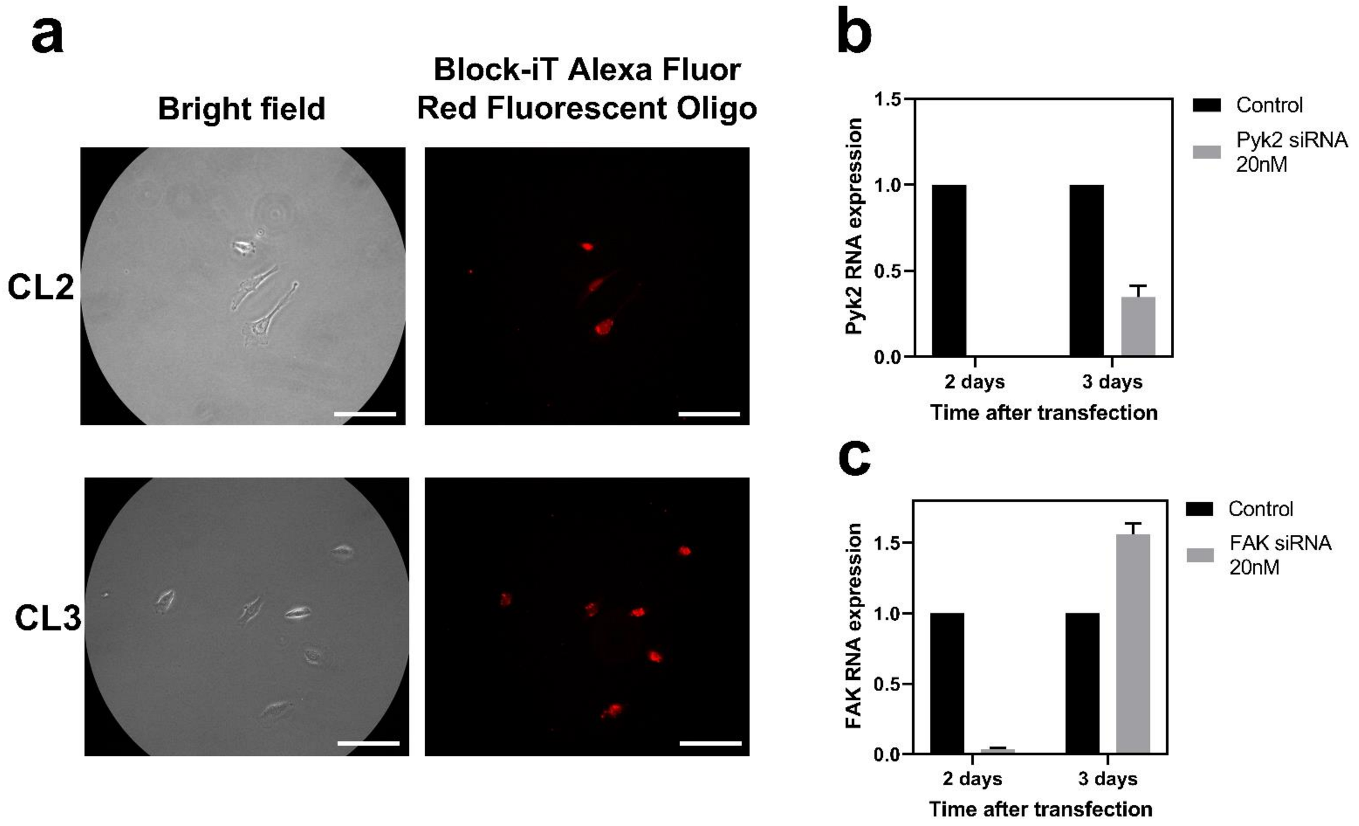

Figure S5. Efficacy of siRNA knockdown against Pyk2 and FAK in GBM derived primary cells. (**a**) Fluorescent microscopy images of CL2 and CL3 transfected with Bock-iT Alexa Fluor red Fluorescent Oligo (20 nM) and Lipofectamine RNAiMAX (3 µL). Magnification of 20× is shown. Scale bar, 100 µm. (**b**,**c**) Pyk2 (b) and FAK (c) relative RNA expression levels in primary glioma cells 2 and 3 days after siRNA knockdown against Pyk2 and FAK.

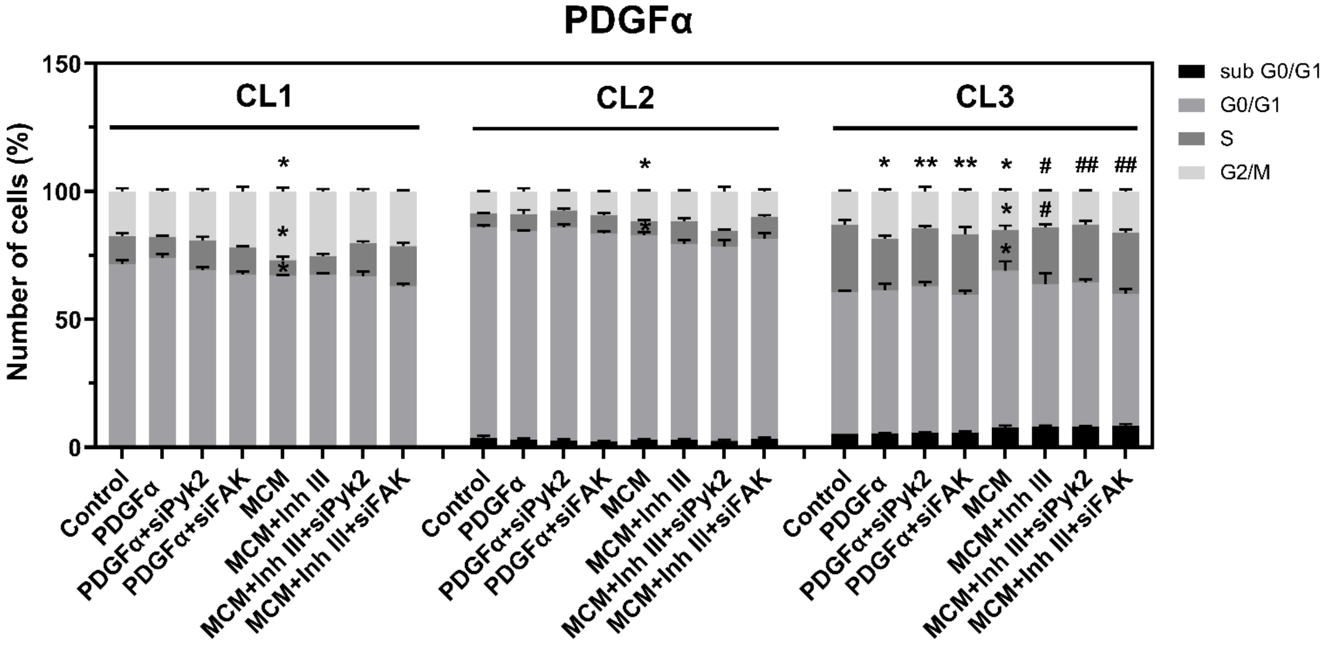

Figure S6. PDGFα-induced cell proliferation in CL3. Cell cycle analysis was performed to evaluate glioma cell proliferation with flow cytometry for GBM-derived primary cells (CL1, CL2, and CL3). The percentage of cells in the sub-G0/G1, G1, S, and G2/M phases was determined by DNA content in PDGFα and Inhibitor III-treated CL1, CL2, and CL3 cells silenced for Pyk2 and FAK expression. The values are shown as means ± SD for 3–6 experiments per group. * $p < 0.05$ vs. control, ** $p < 0.05$ vs. MCM, # $p < 0.05$ vs. PDGFα, ## $p < 0.05$ vs. inhibitor III. Inh III, inhibitor III.
